# Molecular characterization and phylogenetic analysis of small ruminant lentiviruses isolated from Canadian sheep and goats

**DOI:** 10.1186/1743-422X-8-271

**Published:** 2011-06-03

**Authors:** Yvan L'Homme, Mourad Ouardani, Valérie Lévesque, Giuseppe Bertoni, Carole Simard, Giuliano Pisoni

**Affiliations:** 1 Canadian Food Inspection Agency, St-Hyacinthe Laboratory, 3400 Blvd Casavant West, St-Hyacinthe, Quebec, Canada J2S 8E3; 2 Institute of Veterinary Virology, University of Bern, Switzerland; 3 Università degli Studi di Milano, Departement of Animal Pathology, Hygiene and Public Health, via Celoria 10, 20133 Milano, Italy; 4 Department of pathology and microbiology, Faculty of veterinary medicine, University of Montreal, Québec, Canada

## Abstract

**Background:**

Small Ruminant Lentiviruses (SRLV) are widespread in Canadian sheep and goats and represent an important health issue in these animals. There is however no data about the genetic diversity of Caprine Arthritis Encephalitis Virus (CAEV) or *Maedi Visna *Virus (MVV) in this country.

**Findings:**

We performed a molecular and phylogenetic analysis of sheep and goat lentiviruses from a small geographic area in Canada using long sequences from the *gag *region of 30 infected sheep and 36 infected goats originating from 14 different flocks. Pairwise DNA distance and phylogenetic analyses revealed that all SRLV sequences obtained from sheep clustered tightly with prototypical *Maedi visna *sequences from America. Similarly, all SRLV strains obtained from goats clustered tightly with prototypical US CAEV-Cork strain.

**Conclusions:**

The data reported in this study suggests that Canadian and US SRLV strains share common origins. In addition, the molecular data failed to bring to light any evidence of past cross species transmission between sheep and goats, which is consistent with the type of farming practiced in this part of the country where single species flocks predominate and where opportunities of cross species transmissions are proportionately low.

## Introduction

Caprine arthritis-encephalitis virus (CAEV) and ovine *Maedi-visna *virus (MVV) are members of the small ruminant lentiviruses (SRLVs) group in the *retroviridae *family infecting goats and sheep worldwide [1,2]. Lentiviruses from different animal species have in common their genomic organization, the induction of slowly progressive diseases, the large spectrum of targeted organs and symptoms and the ability to persist in their hosts despite a strong immunological response. Transmission of SRLVs is thought to occur predominantly through ingestion of infected milk but, at least in sheep, horizontal transmission may also play a prominent role [3,4]. Common clinical signs caused by SRLV infections include neurological disorders, dyspnoea, emaciation, mastitis and arthritis [2,5,6]. The genomic organization of SRLVs is typical of lentiviruses: positive sense RNA dimmers of approximately 9 kb in size which consist of long terminal repeats (*LTRs*), *gag *(group specific antigens), *pol *(polymerase) *env *(envelope) genes in addition to a number of regulatory genes. The *gag *and *pol *genes are relatively well conserved among SRLVs, which makes them ideal targets for PCR primer design [2]. Originally, MVV and CAEV prototypical strains such as strain K1514 [7], EV-1 [8], SA-OMVV [9] and Cork-CAEV [10] were viewed as distinct viral species restricted to their respective hosts. Viruses isolated from sheep were closely related and referred to as MVV, and those isolated from goats were referred to as CAEV. Over the last two decades however, as more SRLV sequences became available for phylogenetic analyses, it became evident that SRLVs can cross the species barrier since some ovine and caprine strains appear on shared branches in family trees [11-13]. Additionally, molecular-epidemiological evidence suggest that SRLVs can transmit between sheep and goats under favourable conditions (Shah et al 2004; Pisoni et al 2005). In Canada, SRLV infections are widespread in small ruminants and have been associated principally with lung and mammary lesions in sheep and arthritis and emaciation in goats [14,15]. National surveys revealed that 63% of sheep flocks and 52.9% of goat flocks had at least one infected animal ([15];Simard C., unpublished observations). In addition, SRLV were detected in 31.3% of Quebec sheep and 82.5% of milking goats ([16] and unpublished). Despite the fact that SRLVs have been known to circulate and cause disease in Canadian sheep and goats for more than three decades, molecular characterization of SRLV strains had never been carried out in either species [17]. In this study, we report for the first time, genomic sequences and phylogenetic analyses of Canadian SRLVs from a small geographic area. Nearly complete *gag *sequences from both sheep and goats were obtained from animals belonging to single species flocks.

## Materials and methods

A total of 139 goats and 101 sheep originating from 9 and 5 flocks respectively, were investigated in the present study between 2004 and 2006. The study was approved by the CFIA animal ethical committee. All samples were tested for the presence of SRLV serum antibodies using a recombinant ELISA [18] and by nested PCR. All flocks were single species flocks. WBC were isolated from whole blood and DNA was extracted using the QIAamp DNA blood kits following the manufacturer's instructions (Qiagen, Mississauga, Ont). We designed primers corresponding to the most highly conserved sequences in the gag/pol regions from available SRLV genomes in public databases. A total of 6 MVV and 6 CAEV primers were chosen which amplified 1211 bp and 1250 bp fragments respectively (Table 1). Four primers were used in the first PCRs to increase the probability of amplifying the targeted viral segments, and 2 primers were used in the second nested PCRs. The general conditions for PCR were as follows: between 0.5-1 μg total DNA/reaction, 1X PCR buffer (Invitrogen, Mississauga, ON), 200 μM of each dATP, dCTP, dGTP and dTTP (Invitrogen, Mississauga, ON), 250 nM of each primer, 3 mM MgCl_2_, and 1U *Platinum Taq *DNA polymerase (Invitrogen, Mississauga, ON). Final reaction volume was 50 μl. Activation of *Platinum Taq *and initial denaturation was done at 94°C for 2 minutes followed by 35 cycles at 94°C for 1 minute, 55°C for 1 minute and 72°C for 2 minutes. Nested amplifications were carried out in the same conditions as the first round of PCR with 1 μl from PCR reaction 1. Detection of amplicons was carried out on 1% agarose gel electrophoresis and SYBRsafe staining (Invitrogen, Mississauga, ON). PCR products were purified on PCR cleanup columns (Qiagen, Mississauga, ON) and cloned using the PCR TOPO 2.1 (T/A) cloning kit (Invitrogen, Mississauga, ON). Each gag fragment was cloned and 3 clones of each strain were sequenced in both directions, the consensus sequence was used in the aligments. Sequencing reactions were performed using Big Dye v3.1 chemistry from Applied Biosystems on a 3730 × l sequencer (Applied Biosystems). Nucleotide sequences were analysed and edited using the BioEdit software v 7.0.9.0. Multiple alignments of the nucleotide sequences and the deduced amino acid sequences were generated with ClustalW built in MEGA 4.1 [19]. Mean pairwise genetic distances were calculated using MEGA version 4.1 with the *p*-distance model, applying the default setting and with the exception that all sites with ambiguous codes and gaps were ignored. Two phylogenetic analyses were performed. First, Canadian SRLV nucleotide sequences and representative SRLV strains belonging to different SRLV group A and B according to the classification of Shah et al [13] were compared. Second, Canadian SRLV sequences, reference SRLV strains and field strains covering different geographic origin were aligned. Phylogeny reconstruction was carried out using the Neighbour-Joining (NJ) method [20] implemented in MEGA 4.1 with the Tamura-Nei gamma distance. The statistical confidence of the topologies was assessed with 1000 bootstrap replicates. The shape parameter alpha for a discrete gamma distribution of substitution rates, which accommodates for substitution rate variation across sites (e.g. higher substitution rates in mutational hot spots; [21] and the transition/transversion rate ratio parameter kappa were estimated simultaneously by maximum likelihood by using TREE-PUZZLE [22]. The substitution model assumed was Felsenstein's F84 model which accommodates unequal base frequencies and transitions/transversion rate bias [23].

**Table 1:** List of primers used in this study (5' to 3')

Name	Sequence	Location^a^	PCR^b^
CAEV F0	AACTGAAACTTCGGGGACGCCTG	305-327	1

CAEV F1	AAGGTAAGTGACTCTGCTGTACGC	334-357	1

CAEV F2	TGGTGAGTCTAGATAGAGACATGG	513-536	2

CAEV R0	GTTATCTCGTCCTAATACTTCTACTGG	2092-2118	1

CAEV R1	TTTTTCTCCTTCTACTATTCCYCC	2000-2024	1

CAEV R2	GGACGGCACCACACGTAKCCC	1820-1840	2

MVV F0	AAGTAAGGTAAGAGAGACACCTACTGG	539-566	1

MVV F1	TAGATAGAGACATGGCGAAGCAAGCTC	722-749	1

MVV F2	GACAGAAGGGAACTGTCTATGGGC	834-857	2

MVV R0	GGTGGTGCTTCTGTTACAACATAGG	2059-2083	1

MVV R1	GGACGGCACCACACGTGG	2035-2052	1

MVV R2	CCCCTCCTGYTGTTTCCCTG	2014-2034	2

^a ^Location number according to CAEV-Cork isolate [M33677] for CAEV primers and MV US 85/34 isolate [AY101611] for MVV primers

^b ^Used in first PCR (1) or second "nested" PCR (2)

## Results and discussion

**Figure 1:**
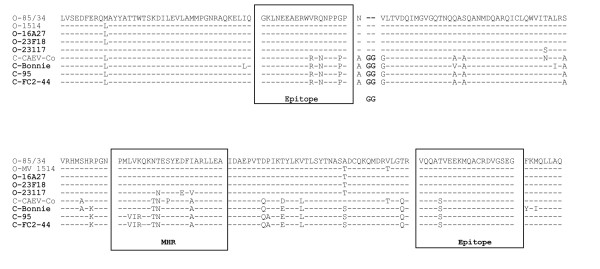
**Amino acid sequence alignment of selected SRLV strains from this study (bold) and reference strains (MV-85/34, MV1514, CAEV-Co) from sheep (O) and goats (C)**. Identical residues are indicated by a dash (-). The conserved MHR (Major Homology Region) and the two immunodominant epitopes are boxed.

We amplified genomic fragments of approximately 1200 nucleotides representing 90% of the *gag *gene (corresponding to position 600-1800 nt region of prototype CAEV-CO, M33677) from 36 goats and 30 sheep from 14 different single-species flocks. Intra-animal mean nucleotide divergence ranged from 0.4 to 1% in sheep and from 0.2 to 0.9% in goats. Intra-farm mean nucleotide divergence ranged from 0.5 to 2% in sheep and from 3.5 to 6.4% in goats. For simplicity, we selected representative strains from each flock, 9 from goats and 5 from sheep for phylogenetic analyses. Canadian SRLV gag sequences from sheep and goats were aligned with and compared to prototypical SRLV sequences. Results show that SRLVs isolated from goats contained a double glycine "**GG**" motif (in bold in figure 1) characteristic of the CAEV-Cork lineage and were most similar to prototypical CAEV strains. SRLV sequences isolated from sheep did not contain the "GG" motif and were most similar to prototypical MVV strains (figure 1). The mean percentages of divergence at the nucleotide level among proviral sequences from goats and sheep were 8.1 ± 0.5% and 4.5 ± 0.4%, respectively; while the mean percentage of divergence between sequences from the two species was 25.5 ± 1.2%.

**Figure 2:**
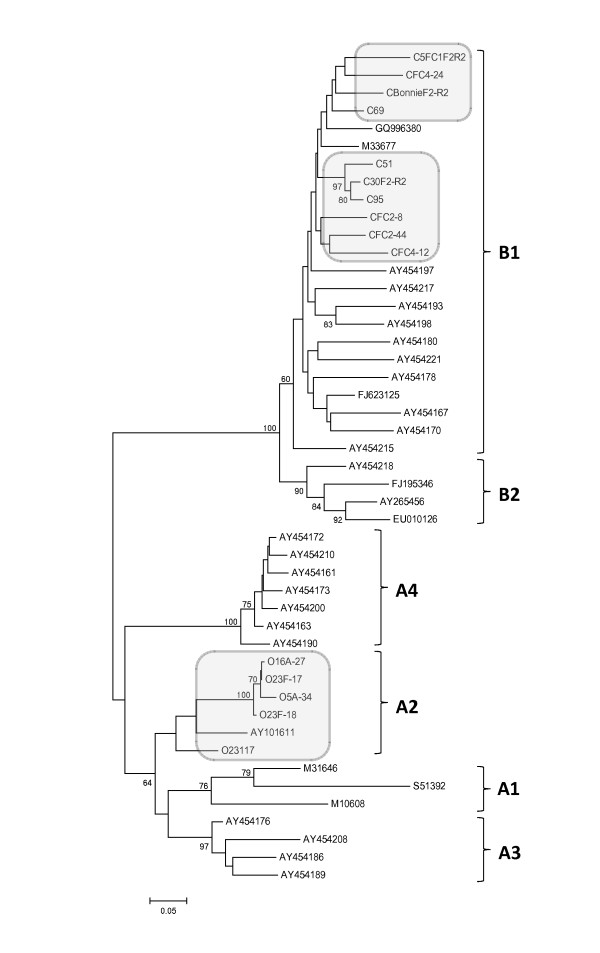
**Phylogenetic relationship of goat and sheep lentiviruses nucleotide sequences isolated from Canadian flocks**. The sequences are ~650 bp comprising 45% of the *gag *gene (corresponding to the 1150-1800 nt region of prototype CAEV-CO, M33677). The unrooted tree was constructed by the neighbour joining method, as described under methods. Bootstrap values are based on 1000 repetitions and are shown at the nodes. SRLV isolates of the present investigation are shown together with available database sequences originating from different geographical areas. Canadian SRLV sequences are highlighted by grey areas. Sequences from the present study are labelled with O or C representing ovine or caprine isolates, respectively. Database derived sequences are denoted with their GenBank accession number. All SRLV sequences characterized in this study are available under accession number [GenBank: HQ158122 to HQ158136]

**Figure 3:**
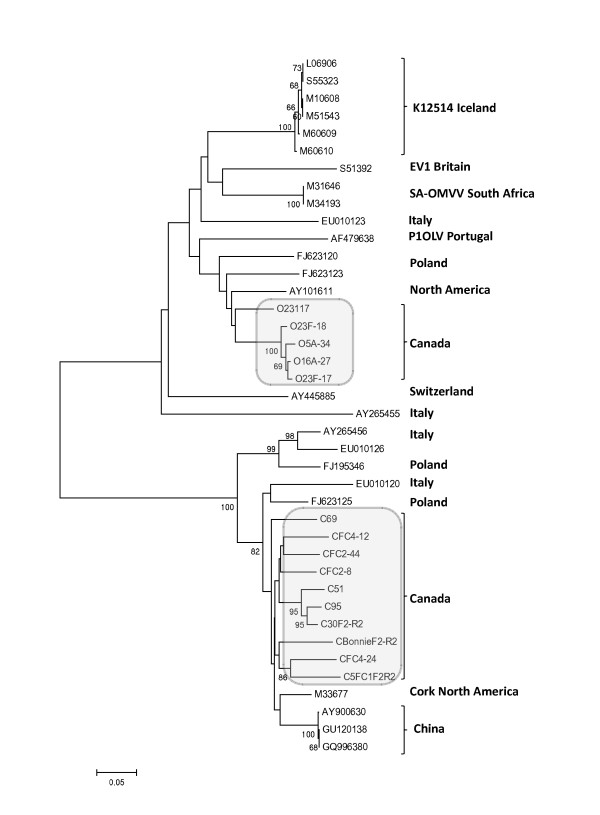
**Phylogenetic relationship of goat and sheep lentiviruses nucleotide sequences isolated from Canadian flocks**. The sequences are ~1200 bp comprising 90% of the *gag *gene (corresponding to the 600-1800 nt region of prototype CAEV-CO, M33677). The unrooted tree was constructed by the neighbour joining method, as described in methods. Bootstrap values are based on 1000 repetitions. SRLV isolates of the present investigation are shown together with available database sequences belonging to the SRLV groups A and B according to the classification of Shah et al., 2004 [13]. Canadian SRLV sequences are highlighted by grey areas. Sequences from the present study are labelled with O or C representing ovine or caprine isolates, respectively. Database derived sequences are denoted with their GenBank accession number. All SRLV sequences characterized in this study are available under accession number [GenBank: HQ158122 to HQ158136]

Multiple alignments of prototypical SRLV strains belonging to different SRLV group A (A1, A2, A3 and A4) and B (B1 and B2) along with Canadian SRLV sequences were performed using approximately 45% of the gag gene (~650 bp corresponding to position 1150-1800 nt of the prototype CAEV-CO, M33677). From these sequence alignments, the gamma distribution parameter alpha and the transition/tranversion ratio were calculated as 0.28 (S.E. 0.01) and 3.05 (S.E. 0.18), respectively. A phylogenetic tree was built using these alignments as shown in figure 2. The caprine proviral isolates tightly segregate with CAEV-like sequences belonging to SRLV subtype B1 together with the CAEV-Cork prototype strain isolated from a North American goat. Mean nucleotide divergence between Canadian caprine and CAEV-Cork sequence is 7.8 ± 0.6%. Ovine proviral isolates were most similar to MVV-like sequences belonging to SRLV subtype A2 clustering with *visna *virus strain 85/34 isolated from a North American sheep. Mean nucleotide divergence between Canadian ovine and strain 85/34 sequences was 9.8 ± 0.8%. Multiple alignments of reference and field SRLV strains from different geographic origin including Canadian strains using the entire sequenced nucleotides fragments (~1200 bp - 90% of the gag gene from position 600 nt to1800 nt of prototype strain CAEV-CO, M33677) were also performed. From these alignments, the gamma distribution parameter alpha and the transition/tranversion ratio were calculated as 0.39 (S.E. 0.02) and 3.11 (S.E. 0.14), respectively. The phylogenetic reconstruction using this alignment (figure 3) confirms the close clustering of caprine and ovine strains from this country with CAEV-like and MVV-like sequences, respectively. Furthermore, according to the geographic origin of SRLV strains, Canadian and North America SLRV sequences appear closely related suggesting a common origin. No Canadian strains were found to cluster in "mixed" SRLV groups of strains detected in sheep and goats such as groups A3 or A4 in the phylograms.

Before the mid 90s, as the first genomic sequences became available, SRLVs from distinct geographical origins were grouped according to the species they were isolated from. Two distinct but related taxonomic clusters were evident, one evolving in sheep and the other evolving in goats. In the mid 90s however, as more sequences from SRLVs were characterized, more complex distribution of strains became evident upon phylogenetic reconstructions, suggesting that SRLVs could no longer be grouped solely based on the species they were obtained from. These findings modified the perception about the faithful relationship between SRLVs and their hosts and led to the concept of cross species transmission [11]. Numerous phylogenetic studies followed and brought additional evidence that SRLVs could no longer be classified according to the species they were recovered from [13,24]. Nowadays, MVV and CAEV are no longer viewed as two distinct viral species infecting exclusively sheep and goats, respectively, but are rather viewed as a continuum of strains or quasispecies that can transmit between small ruminants under favourable conditions [1,11,25].

Results from our study somehow contrast with these recent studies which have generally reported more complex phylogenetic relationships between SRLVs and their hosts. The relatively homogeneous strain populations that were found within each host species in our study could be explained by a founder effect coupled to the single species flock type of management that prevails in this part of the country. Single species flocks type of farming obviously limits close contact between the two species and diminishes the risks of cross species transmission. SRLV strains confined to a single species are expected to be more homogeneous than if transmitted between different host species with the need to readapt each time. Alternatively, our small sample size might under represent the true variety of SRLV strains circulating in small ruminants of this country. A further explanation could come from the primers used in our study, which might have selected specific strain subgroups. Although we think of this last possibility as being highly unlikely since we used combinations of degenerate primers from conserved sequences, it cannot be completely ruled out. Future large-scale studies including more flocks from different geographical regions and breeds in addition to mixed flocks are warranted and might unveil a more thorough picture of the strain diversity present in the small ruminant population of this country.

All novel SRLV sequences reported in this study are available in [GenBank: HQ158122 - HQ158136]

## Competing interests

The authors declare that they have no competing interests.

## Authors' contributions

YL conceived the study, its design and coordination and drafted the manuscript. MO carried out all molecular work. VL carried out the immunoassays. CS helped design the study and helped draft the manuscript. GB helped draft the manuscript. GP carried out the phylogenetic analyses and helped draft the manuscript. All authors read and approved the final manuscript.
